# Simultaneous *in vivo* imaging of Ca^2^^+^ signals in periarteriolar cholinergic axonal varicosities and arteriolar diameter changes in the mouse cerebral cortex

**DOI:** 10.1016/j.jphyss.2026.100094

**Published:** 2026-08-19

**Authors:** Nobuhiro Watanabe, Harumi Hotta

**Affiliations:** Department of Autonomic Neuroscience, Tokyo Metropolitan Institute for Geriatrics and Gerontology, Japan

**Keywords:** Basal forebrain cholinergic neuron, Axonal varicosity, Cerebral arteriole, Somatosensory stimulation, Two-photon imaging, *In vivo*

## Abstract

Basal forebrain cholinergic neurons project widely to the cerebral cortex and participate in cerebrovascular regulation. Although cholinergic axons are distributed around the cerebrovasculature, their functional relationship with arteriolar dynamics remains unclear. In this study, we established an *in vivo* two-photon imaging approach to simultaneously measure Ca²⁺ signals in cholinergic axonal varicosities and arteriolar diameters in urethane-anesthetized mice. An adeno-associated virus (AAV) vector (rAAV-ChAT-jGCaMP8s) was injected into the nucleus basalis of Meynert. *In vivo* imaging of the frontal cortex revealed bead-shaped GCaMP signals around the arterioles. Pinch stimulation transiently increased Ca²⁺ signals in periarteriolar varicosities, followed by arteriolar dilation, with an approximately 2-s delay between their peaks. Linear regression analysis disclosed a significant relationship between the magnitudes of these changes. This approach enabled simultaneous evaluation of cholinergic axonal activity and arteriolar dynamics *in vivo*, providing a tool to investigate the cholinergic regulation of cerebrovasculature.

## Highlights

• AAV-ChAT-GCaMP enables selective imaging of cholinergic projections
• Two-photon imaging reveals bead-shaped Ca^2+^ signals around arterioles
• Sensory stimulation increases periarteriolar cholinergic axonal Ca^2+^ signals
• Axonal Ca^2+^ signals are associated with arteriole dilation

## Graphical abstract

**Figure:**
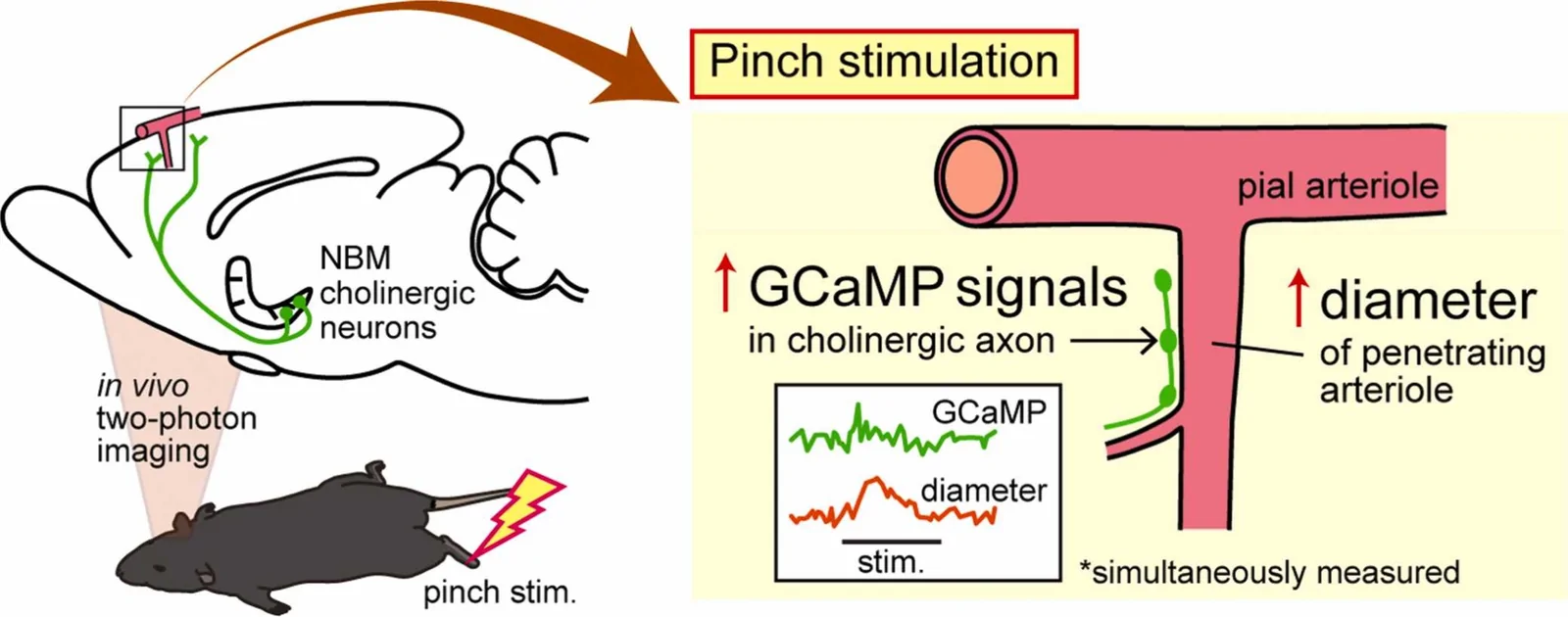

## Introduction

Cholinergic neurons in the basal forebrain constitute a crucial neural system responsible for cognitive function. These neurons project axons to widespread brain regions such as the cerebral cortex, hippocampus, and olfactory bulb [1], [2]. These neurons release acetylcholine (ACh), which contributes to attention, learning, and memory as a neuromodulator [3]. Clinical studies revealed the loss of neurons in the nucleus basalis of Meynert (NBM) within the basal forebrain in patients with Alzheimer’s disease [4], [5]. Thus, cholinergic neurons remain important targets of investigation [6].

In addition to their neuromodulatory role, cholinergic neurons in the basal forebrain help regulate cerebral blood flow (CBF) as arteriole dilators [7], [8], [9], [10], [11]. The cholinergic system is involved in CBF regulation in response to somatosensory stimulation [12], [13], [14], [15]. For example, noxious skin pinch stimulation excites approximately 80% of basal forebrain neurons [16]. Additionally, pinch stimulation increases cerebral ACh release [17] and blood flow [18]. Although such noxious somatosensory stimulation affects systemic blood pressure and heart rate, and the stimulation-induced CBF increases persist even after suppressing the systemic cardiovascular responses, the remained CBF responses are attenuated by pharmacological blockade of ACh receptors [19], [20], [21]. Based on these accumulated lines of knowledge, it has been proposed that skin stimulation excites cholinergic neurons in the basal forebrain, increases ACh release, and dilates the arterioles.

In cholinergic neurons, ACh is released from varicosities, which are bead-shaped axonal regions containing synapse vesicles [22], [23], [24]. Cholinergic varicosity activity is monitored *in vivo* by two-photon imaging of Ca^2^^+^ signals using a genetically encoded Ca^2^^+^ sensor (i.e., GCaMP) expressed in basal forebrain cholinergic neurons via adeno-associated virus (AAV) vectors [25], [26], [27], [28]. Additionally, a study using these methods revealed that an increase in axonal Ca^2^^+^ signaling precedes ACh release [29]. Histologically, the axonal varicosities of basal forebrain cholinergic neurons are distributed around blood vessels in the cerebral cortex [24]. However, the functional relationship between cholinergic axonal Ca^2+^ signals at periarteriolar varicosities and arteriolar responses has not been directly examined. Thus, we hypothesized that simultaneous *in vivo* imaging of arterioles and periarteriolar cholinergic axonal varicosities would reveal their functional relationship during somatosensory stimulation in a single experiment setting.

In the present study, we established an *in vivo* imaging method to simultaneously measure cerebral arteriolar diameter and Ca^2^^+^ signals in periarteriolar cholinergic axonal varicosities by expressing GCaMP in basal forebrain cholinergic neurons using an AAV vector carrying the choline acetyltransferase (ChAT) promoter [30] and labeling blood plasma using fluorescent dye [9], [31]. Using this approach, we further examined the relationship between periarteriolar varicosity activity and arteriolar dilation during skin pinch stimulation.

## Materials and methods

### Animals

Eight male C57BL/6 J mice (7–12 months old; weight, 28.3–30.9 g) were used. The animals were purchased from Japan Charles River (Yokohama, Japan) and housed at the Tokyo Metropolitan Institute for Geriatrics and Gerontology. They were housed in individually-ventilated cages with free access to a commercial pelleted diet (CRF−1, Oriental Yeast Co., Ltd., Tokyo, Japan) and filtered tap water containing 2 ppm chlorine. The vivarium was maintained at 22°C ± 1°C and 50% ± 10% relative humidity with a 12-h/12-h light/dark cycle (lights off at 20:00 h). The study protocol was approved by the Animal Care and Use Committee (approval number: 22020) and the committee for recombinant DNA experiments (approval number: 188) of the Tokyo Metropolitan Institute for Geriatrics and Gerontology. The protocol additionally followed the “Guidelines for Proper Implementation of Animal Experiments” established by the Science Council of Japan in 2006.

### Pre- and postoperative care for AAV vector injection

To ensure acclimatization to the breeding environment after surgery, gel food (MediGel® Sucralose, ClearH_2_O, Westbrook, ME, USA), a heating pad (Kowa, Tokyo, Japan), and enrichment devices (wooden blocks and paper tunnel) were placed in cage for 3–4 days before surgery.

After the surgery, mice were housed individually until experimentation. The heating pad was kept in cage for the first 2 days after surgery, and gel food containing analgesic (meloxicam, 5 mg/kg/day) was provided alongside regular chow and drinking water for the first week. The enrichment devices were provided through the postoperative period, and the bedding was exchanged every week.

### Surgery for AAV vector injection

To express fluorescent protein in cholinergic neurons originating from the NBM, a ChAT promoter-driven AAV vector [30] was used. rAAV-ChAT-jGCaMP8s was injected into the NBM for two-photon imaging in seven mice (AAV9 serotype, titer = 6.05 × 10^12^ genome copies/mL, BC−4826, packaged by Brain Case, Wuhan, China).

Aseptic surgery was performed under isoflurane anesthesia (initially 4% with room air, followed by 2%–3% during surgery). Prior to any surgical procedures, antibiotics (cefazoline sodium, 50 mg/kg) and analgesics (buprenorphine, 0.03 mg/kg; meloxicam, 5 mg/kg) were subcutaneously injected. The scalp fur was trimmed using conventional clippers and disinfected using povidone–iodine solution (Isodine®, Shionogi Co., Ltd., Osaka, Japan) and 70% isopropyl ethanol. Ointment was applied to the eyes to prevent drying during surgery.

Each animal’s head was fixed to a stereotaxic instrument with ear bars (SR−5 M-S, Narishige, Tokyo, Japan) after local anesthetics were applied to the external ear canals (2% Xylocaine® jelly, Sandoz Pharma K.K., Tokyo, Japan). The scalp was incised along the midline after procaine hydrochloride was subcutaneously injected. The skull was exposed and partly excised using a dental drill, and the dura was punctuated using a 30 G syringe needle. The needle of a Neuros Syringe (7001 KH Neuros Syringe, HAMILTON, Reno, NV, USA) filled with the AAV vector was inserted into the cerebral cortex and slowly moved (10 μm/s) to the right NBM (anteroposterior, −0.9 mm from bregma; lateral, 2.2 mm from midline; ventral, 4.2 mm from the dorsal brain surface; [32]). The needle was further moved to 4.6 mm and slowly returned to the target position. The AAV vector was injected over 18 min at 50 nL/min (total volume, 900 nL) using an infusion pump (UMP3 UltraMicroPump and Micro4 Controller, World Precision Instruments, Sarasota, FL, USA). These procedures for intracerebral injection of AAV vector were referred to the methods described by Okuda et al. [33]. After the injection was completed, the needle was kept in place for 10 min and then slowly removed. The exposed cortical surface was sealed with a piece of plastic wrap (AsahiKASEI, Tokyo, Japan) and covered with dental cement (Inosit Baseliner, DMG Chemisch-Pharmazeutische Fabrik GmbH, Hamburg, Germany). The skin was sewn with a sterile 4–0 nylon suture and fixed with a tissue adhesive (Aron Alpha A, Daiichi Sankyo, Tokyo, Japan). Following surgery, warm saline (500 μL) was subcutaneously injected for body fluid supplementation.

**Fig. 1:**
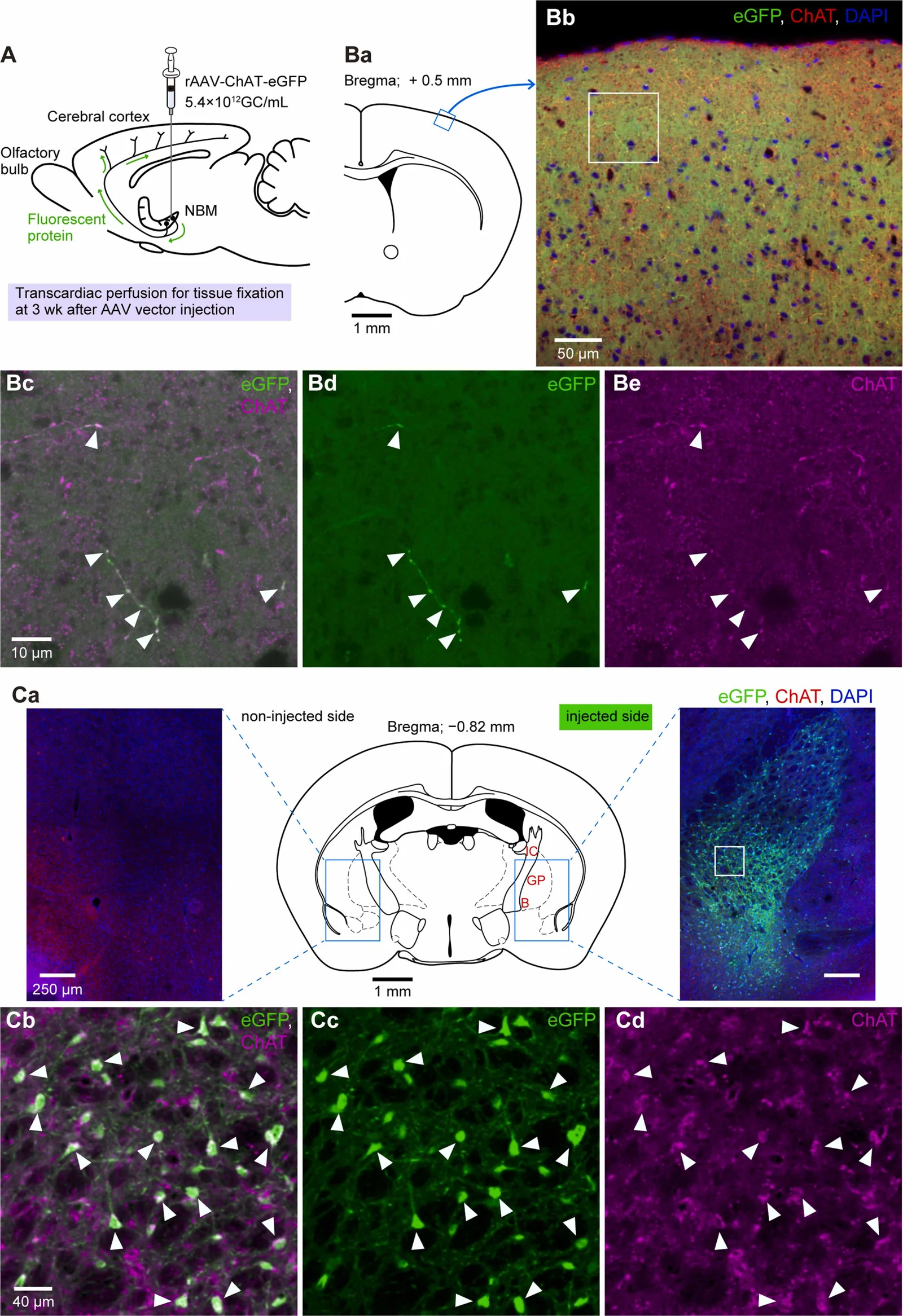
Colocalization of eGFP with ChAT immunoreactivity in the mouse frontal cortex and NBM following rAAV-ChAT-eGFP injection into the NBM. **A**: Schematic diagram of AAV vector injection into the NBM. Animals were transcardially perfused 3 weeks after injection, allowing sufficient anterograde transport of the fluorescent protein into axons originating from the NBM. **B**: Confocal images showing eGFP-expressing axons originating from the NBM, ChAT immunoreactivity (anti-ChAT antibody), and DAPI staining in the frontal cortex. (a) Schematic diagram of a coronal section indicating the confocal imaging area (0.5 mm rostral to bregma). The diagram was drawn based on mouse brain atlas [32]. (b) Confocal image of the frontal cortex, as indicated by the light blue boxed region in (a). (c-e) Enlarged views of the white boxed region in (b). Arrowheads indicate colocalization of eGFP and ChAT immunoreactivity. **C**: Confocal images showing eGFP-expressing neurons, ChAT immunoreactivity (anti-ChAT antibody), and DAPI staining in the NBM. (a) Schematic diagram of a coronal section indicating the confocal imaging area (0.82 mm caudal to bregma) and confocal images, as indicated by the light blue boxed region. B indicates the NBM; GP, globus pallidus; IC, internal capsule. (b-d) Enlarged views of the white boxed region in (a).

In a preliminary experiment, we examined via immunohistochemistry whether the ChAT promoter-driven AAV vector could express a fluorescent protein in cholinergic axon varicosities in the cerebral cortex (Fig. 1). A vector was injected into the right NBM in one mouse (AAV9 serotype, rAAV-ChAT-eGFP, titer = 5.43 × 10^12^ genome copies/mL, BC−4827, packaged by Brain Case). Three weeks after injection (Fig. 1A), the animal was euthanized with isoflurane and transcardinally perfused with PBS and 4% paraformaldehyde in PBS using a peristatic pump. The brain was then removed. Cryosections (40 μm thick) were immunohistochemically stained (details are provided in the Supporting Information). Fig. 1Bb-e presents confocal images of the frontal cortex. eGFP-expressing axons were sparsely observed, and the eGFP signals were colocalized with ChAT immunostaining. Additionally, eGFP-expressing somata were observed in the injected site including the NBM (Fig. 1Ca) and colocalized with ChAT immunostaining (Fig. 1Cb-d). These data suggest that the ChAT promoter-driven AAV vector mediates fluorescent protein expression in cholinergic axons originating from the NBM.

### *In vivo* two-photon imaging (GCaMP and arterioles)

**Fig. 2:**
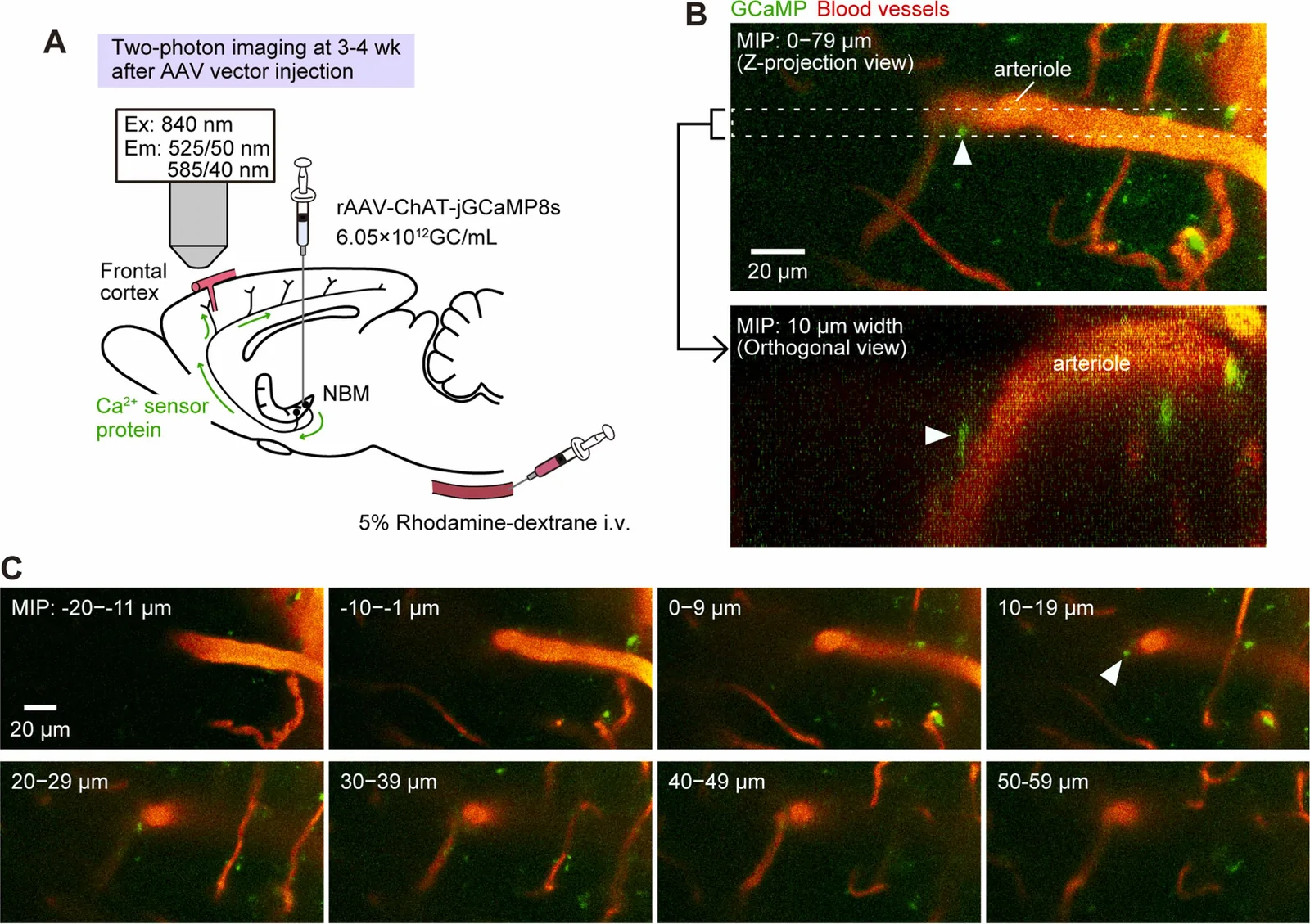
Three-dimensional *in vivo* two-photon imaging of cholinergic axonal Ca^2+^ signals and penetrating arterioles in the frontal cortex. **A**: Schematic diagram for *in vivo* two-photon imaging. Two-photon imaging of the frontal cortex was performed 3–4 weeks after AAV vector injection into the NBM, allowing sufficient anterograde transport of the Ca^2+^ sensor protein into axons of NBM cholinergic neurons. A fluorescent dye (5% rhodamine–dextran) was intravenously injected to visualize blood vessels. **B**: Example of three-dimensional two-photon imaging. The upper panel shows a *Z*-projection view with a maximum-intensity projection (MIP) constructed from *Z*-stacks spanning 80 slices, acquired at 1-μm intervals. The lower panel shows an Orthogonal view, constructed from the region enclosed by the dashed line in the upper panel and displayed as a MIP over a 10-μm width. Arrowheads indicate a periarteriolar varicosity of a cholinergic axon. **C**: Series of *Z*-stack images shown every 10 slices with a MIP.

*In vivo* two-photon imaging was performed 3–4 weeks after AAV vector injection (Fig. 2A). Mice were first anesthetized with isoflurane (4% for approximately 3 min) and then received urethane (1.4 g/kg, subcutaneously). Sufficient depth of anesthesia was determined based on loss of corneal and withdrawal reflexes, and additional anesthetic was administered as needed (injecting 5%–10% of the initial dose of urethane, or inhaling isoflurane). The animals were artificially ventilated through a tracheal cannula (MiniVent Type 845, Harvard Apparatus, MA, USA). Rectal temperature was maintained at 37.0°C–37.5°C using a feedback-controlled heating pad (BWT−100A, Bioresearch Center, Nagoya, Japan), and warm saline was continuously administered through a subcutaneously implanted 30 G needle at 3 μL/min with an infusion pump (ESP−64, EICOM, Kyoto, Japan).

Each animal's head was fixed to a stereotaxic instrument with ear bars. A cranial window (approximately 2 mm in diameter) was made on the frontal cortex by thinning the skull using a dental drill, followed by soaking 10% disodium ethylenediamine tetraacetic acid solution for 10–15 min [31]. Dental cement (Fuji LUTE, GC Corp., Tokyo, Japan) was applied around the cranial window to retain saline for immersing the objective lens. Rhodamine B isothiocyanate–dextran (molecular weight, 70 kDa; Sigma-Aldrich, St. Louis, MO, USA) dissolved in saline (5% solution) was intravenously administered before two-photon imaging at a volume of 80–100 μL. Fluorescence was excited using a two-photon laser (840 nm; Chameleon Vision II, Coherent, Santa Clara, CA, USA) with a × 25 correction collar lens (numerical aperture = 1.00, Leica Microsystems, Wetzlar, Germany). The emission signal was detected on an external detector through bandpass filters (525/50 and 585/40 nm) in the photon counting mode.

The site at which varicosities expressing detectable GCaMP signals were present near an arteriole branching from a pial arteriole originating from the anterior cerebral artery was selected for imaging. To obtain structural information, three-dimensional (*XYZ*) images were obtained. One *XY* plane image consisted of 1024 × 512 or 1024 × 1024 pixels with a pixel size of 0.2 μm, and depth scanning (*Z*) was performed with a step size of 1 μm.

To examine the influence of somatosensory stimulation, time-series images (*XYt*) were acquired for 30 s at a sampling rate of 3.8–7.5 frames/s. Pinch stimulation was applied to the left hind paw for 10 s. The same investigator manually performed the stimulation and attempted to apply a consistent force (approximately 5–6 kg, measured using a Jamar® hydraulic pinch gauge, 5030HPG, J.A. Preston, Jackson, MI, USA). Trials were separated by at least 3 min.

### Analysis of *in vivo* two-photon imaging data

For *XYZ* imaging data, images were analyzed using Imaris software (v 10.0.0, Bitplane, Belfast, UK). The volume and minor radius of cholinergic varicosities and the distance from the closest arteriole were measured using the Surface function of the software. Fluorescence signals of GCaMP and Rhodamine B (arterioles) were smoothed using a Gaussian filter (diameters of 1 and 3 μm, respectively). The binarization threshold was determined by the full width at half-maximal intensity.

For *XYt* imaging data, the fluorescence intensity of GCaMP was measured using the Spots function in Imaris to automatically track fluorescence signal clusters over time. Additionally, imaging plane stability was confirmed based on preserved microvessel architecture. Raw fluorescence (F) was extracted using a region of interest with a diameter of 3–4 μm that sufficiently included each varicosity. Relative fluorescence was calculated as ΔF/F(%) = [(F − F_0_)/F_0_] × 100. F_0_ was estimated by curve fitting to account for gradual baseline drift using exponential, polynomial, or power approximation functions, depending on the curvature before pinch stimulation. The diameter of the arteriole was measured using the Surface function in Imaris or ImageJ software with a plug-in program (Diameter, v 1.0). A 3-μm-diameter Gaussian filter was applied for smoothing. Imaris software was used when binarized arteriole images exhibited an oval shape, whereas ImageJ was used when the images had a non-oval shape. The binarization threshold was determined by the full width at half-maximal intensity in the first frame of each trial, and each frame was visually inspected to ensure proper binarization. Diameter changes were expressed as Δ% relative to the baseline value (the average diameter during the 5 s before stimulation).

The obtained values were imported into Spike 2 software (v10.2.8., Cambridge Electronic Design, Cambridge, England), temporally smoothed using a moving average over three consecutive data points, and then resampled at 3.8 Hz. The peaks of GCaMP signal and arteriolar diameter changes were defined as the maximum changes after pinch stimulation onset. Additionally, cross-correlation analysis was performed to investigate the relationship between GCaMP signal and arteriolar diameter dynamics over time, using a 6-s time window centered on stimulation onset. The arteriolar diameter trace was shifted every one frame (0.263 s), and Pearson’s correlation coefficients were calculated at each shift by Excel (Microsoft Office 2016).

### Statistical analysis

Statistical analyses were performed using Prism software (version 9.5.1, GraphPad Software Inc., Boston, MA, USA). The times to peak GCaMP signals and arteriolar diameter were compared using the paired *t*-test based on the normality of data, as tested using the Shapiro–Wilk test. The relationship between the magnitudes of the GCaMP signal and arteriolar diameter changes was evaluated using simple linear regression analysis. The peak correlation coefficient was tested against zero using a one-sample *t*-test. p < 0.05 indicated statistical significance. Data are expressed as the range (minimum-maximum) or median [interquartile range (IQR)].

## Results and discussion

### Two-photon imaging of GCaMP signals in the varicosities of cholinergic axons and blood vessels in the frontal cortex

We explored the cerebral parenchyma and arterioles to observe GCaMP signals near arterioles in the frontal cortex using two-photon microscopy (Fig. 2A). An example of three-dimensional imaging is presented as a maximum-intensity projection in Fig. 2B and C, and bead-shaped GCaMP signals were found around the arterioles (indicated by arrowheads), near capillaries, and within the cerebral parenchyma. Orthogonal views (Fig. 2B, lower panel) and a series of horizontal images along the *Z*-axis (Fig. 2C) indicated that a periarteriolar bead-shaped GCaMP signal was located approximately 15 μm below the cortical surface.

Among the seven mice, six completed the two-photon imaging experiment, whereas one died during the experiment (cause unspecified). At least one periarteriolar bead-shaped GCaMP signal was detected in each of the six surviving mice, yielding a total of eight bead-shaped GCaMP signals (Table 1). Of these eight bead-shaped GCaMP signals, six were observed around penetrating arterioles at depths of 5–40 μm from the cortical surface, and two were found around an arteriole at the cortical surface level before it entered the brain parenchyma. These GCaMP signals were located at a site 0.33–4.89 μm from the nearest arteriole (lumen diameter, 9–16 μm). Considering the thickness of the arteriolar wall (approximately 10% of the lumen diameter [34]), the bead-shaped GCaMP signals were estimated to be within 3 μm of the outer arteriolar wall surface, consistent with perivascular varicosities reported in previous histological studies [24], [35]. An immunohistochemical study using three-dimensional reconstruction of confocal images showed that cholinergic terminals in the cerebral cortex may contact arteriolar smooth muscle cells and basement membranes [36]. Thus, the bead-shaped GCaMP signals observed in the present study likely represent periarteriolar varicosities of cholinergic axons originating from the NBM. However, owing to the optical resolution [37] and physiological movements such as vasomotion and respiration [38], the measured distances should be considered estimates of spatial proximity rather than precise ultrastructural distances.

**Table 1:** Profile of each periarteriolar varicosity of cholinergic axons.

Varicosity #	Mouse #	Varicosity diameter (μm)	Depth of periarteriolar GCaMP signal location (μm)	Maximum depth at which GCaMP signals were detectable (μm)	Distance between varicosity and arteriole (μm)	Arteriolar diameter (μm)
1	M#1	1.97	40	42	2.79	13.8
2	M#1	4.24	12	42	4.89	15.9
3	M#2	2.77	13	15	0.33	12.9
4	M#3	4.71	0	50	0.93	13.8
5	M#3	4.17	0	50	4.21	11.3
6	M#4	2.31	15	79	2.37	13.4
7	M#5	3.3	5	27	2.25	9.1
8	M#6	1.48	22	90	2.31	14.9

In total, eight periarteriolar varicosities were detected in six mice. Two varicosities were observed in mice #1 and 3, and one varicosity was detected in the other mice. The depth of the GCaMP signal location represents the distance from the cortical surface. The binarization threshold was determined by using the full width at half-maximal intensity for both cholinergic varicosities and arterioles. The distance between the varicosity and arteriole was defined as the shortest distance between the surface of a cholinergic varicosity and the arteriolar lumen.

The present study focused on two-photon imaging of the superficial cortical layers, based on a previous study reporting that basal forebrain stimulation produced the most robust vasodilatory effect on the superficial layer in mice [9]. Although the layer dependence of GCaMP-expressing varicosities was not determined in the present study, the previously reported dense cholinergic innervation of the superficial cortical layer [2], [39], [40] is consistent with our detection of periarteriolar GCaMP-expressing varicosities in the superficial cortex by two-photon microscopy.

### Temporal relationship between cholinergic varicosity Ca^2+^ signals and arteriolar diameter changes during pinch stimulation

**Fig. 3:**
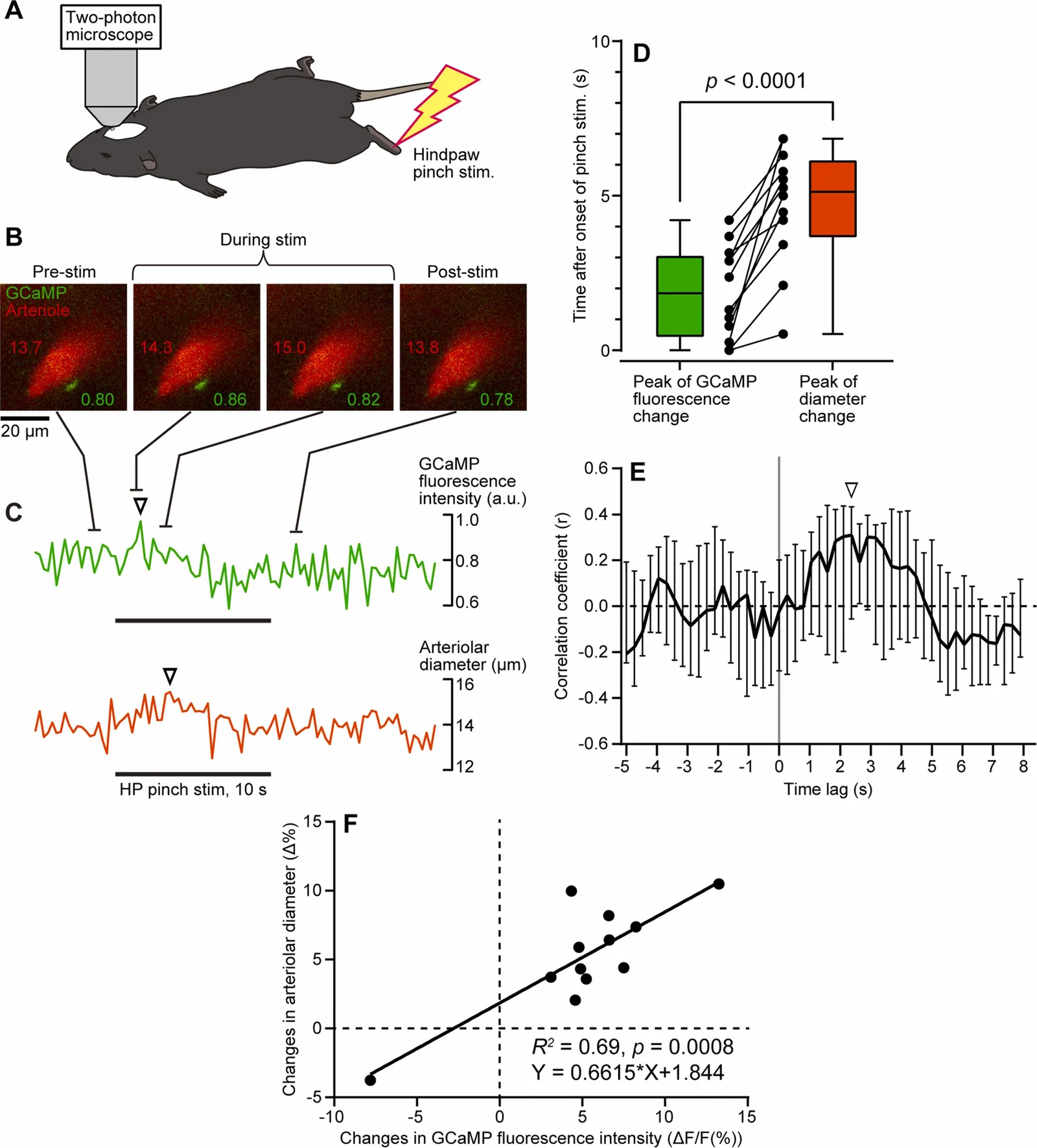
Relationship between periarteriolar cholinergic varicosity Ca^2+^ signals and arteriolar diameter changes in responses to pinch stimulation. **A**: Schematic diagram of pinch stimulation applied to the hindpaw during *in vivo* two-photon imaging. **B**: Example images of a penetrating arteriole and periarteriolar GCaMP-expressing varicosity from a single trial (three consecutive images were averaged; effective temporal resolution, 0.789 s). Numerical values shown on each image are the GCaMP fluorescent intensity (a.u., arbitrary units; in green) and arteriolar luminal diameter (μm; in red). **C**: Time course of changes in GCaMP fluorescence intensity and arteriolar luminal diameter measured at 3.8 Hz (0.263 s of temporal resolution). Pinch stimulation was applied to the left hindpaw (HP) for 10 s. The thick horizontal line indicates the period of pinch stimulation. The thin horizontal lines indicate the time points corresponding to the images shown in panel **B**. Open triangles indicate the peaks of GCaMP fluorescence intensity and arteriolar diameter changes during pinch stimulation. **D**: Temporal relationship between GCaMP signal and arteriolar diameter changes. The time to peak after the onset of pinch stimulation was compared using the paired *t*-test. Individual closed circles connected by lines indicate data obtained from a single trial (n = 12 trials; 1–2 trials/varicosity). Boxes represent the median and interquartile range, and whiskers indicate the minimum and maximum values. **E**: Cross-correlation analysis performed on the time courses of GCaMP fluorescence intensity and arteriolar diameter changes over a 6-s window encompassing 3 s before and 3 s after the onset of pinch stimulation. Positive lag indicates that changes in GCaMP fluorescence preceded arteriolar diameter changes. Correlation coefficient is expressed as the median and interquartile range. An open triangle indicates the peak correlation coefficient (r). **F**: Relationship between the peak amplitudes of GCaMP fluorescence intensity and arteriolar diameter changes (ΔF/F(%) and Δ%, respectively) was analyzed by linear regression. Individual closed circles represent data from individual trials.

Changes in GCaMP fluorescence intensity in the periarteriolar varicosities of cholinergic axons and arteriolar diameter were examined during pinch stimulation (Fig. 3A). Pinch stimulation was applied for 10 s following a 10-s prestimulation (baseline) period. GCaMP fluorescence transiently increased approximately 1 s after stimulation onset (Fig. 3B, C), whereas arteriolar diameter increased and peaked approximately 3 s after stimulation onset before gradually declining toward the baseline level.

Fig. 3D summarizes the temporal relationship between GCaMP fluorescence and arteriolar diameter changes (n = 12 trials; 1–2 trials/varicosity). Peak GCaMP fluorescence occurred shortly after pinch stimulation onset [1.8 s (IQR, 0.39–3.1 s)], whereas peak arteriolar diameter change occurred later at 5.1 s (IQR, 3.6–6.2 s). The paired *t*-test showed that the peak GCaMP fluorescence change preceded the peak arteriolar diameter change by 2.2 s (IQR, 2.1–3.8 s; p < 0.0001). Additionally, cross-correlation analysis revealed that the correlation coefficient peaked at a positive time lag of 2.37 s [r = 0.31 (IQR, −0.056 to +0.43), Fig. 3E]. This peak correlation coefficient was significantly greater than zero (p = 0.046). These findings indicate that GCaMP signal changes over time preceded arteriolar diameter changes, consistent with the difference in peak latency between these measures.

Regarding the temporal relationship between NBM activation and arteriolar dilation, Hotta et al. [9] reported a similar result: the latency of parenchymal arteriolar dilation was < 2.5 s after NBM neuron activation by local electrical stimulation, supporting the validity of the present findings. The present experimental approach enables simultaneous measurement of periarteriolar cholinergic varicosity activity and arteriolar diameter changes during somatosensory stimulation in a single experimental setting, which have previously been studied separately [16], [17], [18].

Separate subanalyses of the non-stimulated baseline and stimulation periods revealed a significant positive correlation during stimulation, but not during the non-stimulated baseline period (Supporting Information Figure S1). In contrast, cortical GABAergic neurons have been reported to contribute to spontaneous arteriolar dynamics, but not to arteriolar responses to sustained somatosensory stimulation [41]. These findings indicate that different neuronal populations may contribute differently to spontaneous and stimulation-evoked arteriolar dynamics. The present experimental approach may provide a useful platform for investigating complex neurogenic regulation of cerebrovascular function.

### Relationship between the amplitudes of cholinergic varicosity Ca^2+^ signals and arteriolar diameter changes

Fig. 3F summarizes the relationship between the amplitude of the GCaMP fluorescence intensity signal and arteriolar diameter changes during pinch stimulation. GCaMP fluorescence increased following stimulation in all but one trial, and arteriolar diameter changes were concordant with GCaMP signal changes. Simple linear regression analysis illustrated that GCaMP fluorescence changes during pinch stimulation were significantly associated with arteriolar diameter changes (R^2^ = 0.69, p = 0.0008; Fig. 3F). This relationship might be supported by NBM stimulation frequency-dependent increases in cerebral ACh release and CBF [10], [42], [43]. In a few trials (two trials in each of two mice), pinch stimulation was applied after intraperitoneal administration of the autonomic ganglionic blocker hexamethonium bromide (20 mg/kg [44]), which does not cross the blood–brain barrier, to reduce the influence of systemic cardiovascular responses to the noxious stimulation. The relationship between the GCaMP signals and arteriolar diameter changes was fundamentally similar to that observed in the experiments without hexamethonium (Supporting Information Figure S2), consistent with the results of a previous study on CBF responses to pinch stimulation [18].

Physiologically, changes in cholinergic varicosity activity around the penetrating arterioles in response to somatosensory stimulation are reasonable from the perspective of CBF control. The penetrating arterioles represent a bottleneck for blood perfusion to the capillary network [45]. Although the arteriolar dilation during pinch stimulation in the present study may appear modest (approximately 5.3%), this change would theoretically correspond to an approximately 23% increase in blood flow through the vessel according to Poiseuille’s law, assuming that other hemodynamic factors remain constant. Therefore, periarteriolar cholinergic varicosities might contribute to CBF regulation by controlling the diameter of these arterioles.

Based on the mechanisms underlying vasodilatory responses to basal forebrain activation, as proposed mainly from pharmacological approaches [8], the Ca^2+^ signals observed in the present study, which reflect activation of periarteriolar cholinergic varicosities, may increase local ACh release and thereby induce nitric oxide-dependent arteriolar dilation. Further studies are needed to test this hypothesis directly.

## Conclusion

We established an experimental approach for simultaneously assessing periarteriolar cholinergic axonal varicosity activity and arteriolar dynamics *in vivo* by expressing GCaMP in basal forebrain cholinergic neurons using a ChAT promoter-driven AAV vector. Using this approach, we examined their functional relationship during somatosensory stimulation. Although this approach revealed a temporal and quantitative association between periarteriolar cholinergic axonal varicosities and arteriolar diameter changes, further studies are required to determine causality.

Additionally, this experimental approach might represent a useful tool for investigating the mechanisms of CBF regulation mediated by basal forebrain cholinergic neurons. Because we employed a ChAT promoter-driven AAV vector to express a Ca^2+^ sensor protein in the basal forebrain cholinergic neurons in wild-type animals, this approach could be applicable beyond specific animal strains.

## Author contributions

N.W. and H.H. conceived, designed the research and acquired data. N.W. analyzed data. N.W. and H.H. interpreted data. N.W. drafted the paper with inputs from co-author and H.H. revised it critically for important intellectual content. N.W. and H.H. approved the final version of the manuscript to be submitted.

## CRediT authorship contribution statement

**Harumi Hotta:** Writing – review & editing, Visualization, Validation, Supervision, Resources, Project administration, Methodology, Investigation, Funding acquisition, Data curation, Conceptualization. **Nobuhiro Watanabe:** Writing – review & editing, Writing – original draft, Visualization, Validation, Resources, Methodology, Investigation, Formal analysis, Data curation, Conceptualization.

## Declaration of Competing Interest

The authors declare the following financial interests/personal relationships which may be considered as potential competing interests: Harumi Hotta reports financial support was provided by Tsumura and Co. Harumi Hotta serves as an Associate Editor of the Journal of Physiological Sciences. Given her role, she had no involvement in the peer review of this article and had no access to information regarding its peer review. Full responsibility for the editorial process for this article was delegated to another journal editor. The other authors declare that they have no known competing financial interests or personal relationships that could have appeared to influence the work reported in this paper. If there are other authors, they declare that they have no known competing financial interests or personal relationships that could have appeared to influence the work reported in this paper.

## Data Availability

The data supporting the findings of this study are available from the corresponding author upon reasonable request.
