# Supplementary material for: Simultaneous in vivo imaging of Ca2+ signals in periarteriolar cholinergic axonal varicosities and arteriolar diameter changes in the mouse cerebral cortex
Source: J Physiol Sci. 2026 Aug 19;76(3):100094. doi: 10.1016/j.jphyss.2026.100094 (PMC13543906; doi:10.1016/j.jphyss.2026.100094)
Supplement: Supplementary file 1 [file mmc1.docx]

**Supporting Information**

**Simultaneous *in vivo* imaging of Ca^2+^ signals in periarteriolar cholinergic axonal varicosities and arteriolar diameter changes in the mouse cerebral cortex**

**Nobuhiro Watanabe, Harumi Hotta**

**Methods**

***Histological analysis***

The removed brain was postfixed in 4% paraformaldehyde in PBS overnight at 4°C and cryoprotected in sucrose solution (10% sucrose in PBS for 4 h and 20% sucrose in PBS for 24–48 h). After rinsing in PBS, the brain was embedded in OCT compound in a plastic mold (Tissue-Tek^®^, Sakura Finetek Japan Co., Ltd., Tokyo, Japan; Square-S22, Polysciences, Inc., Warrington, PA, USA) and frozen on an aluminum block with liquid nitrogen. The tissue was stored at −80°C until further processing.

The brain was sectioned coronally at a thickness of 40 μm using a cryostat (CM3050S, Leica Microsystems, Wetzlar, Germany) and placed on glass slides. After drying, sections were washed with PBS and blocked in PBS containing 0.1% Triton X-100 and 3% normal donkey serum for 1 h at room temperature. Sections were then incubated with a primary antibody (anti-choline acetyltransferase (ChAT), 1:100, AB144P, Millipore, Burlington, MA, USA; RRID: AB_90661) in blocking solution for 24 h at 4°C. After washing with PBS, the sections were incubated with a secondary antibody (Alexa Fluor 594, 1:200, Thermo Fischer Scientific, Waltham, MA, USA; RRID: AB_2534105) for 2 h at room temperature. After washing with PBS containing 0.02% NaN_3_, sections were mounted using a mounting medium containing 4′,6-diamidino-2-phenylindole (DAPI) (VECTASHIELD PLUS with DAPI, H-2000, Vector Laboratories, Inc., Newark, CA, USA).

The immunohistochemically stained sections were imaged using a confocal microscope (STELLARIS WLL, Leica Microsystems) with ×10 HC PL APO CS2 (NA = 0.4), ×20 HC PL APO CS2 (NA = 0.75), and ×40 HC PL APO (NA =1.30, oil CS2) objectives, a digital zoom of ×0.75–3.88 and sequential line scanning for each channel. Colocalization of eGFP signals with anti-ChAT immunostaining was assessed.

***Pharmacology***

To reduce the influence of systemic cardiovascular responses to pinch stimulation, an autonomic ganglionic blocker, hexamethonium bromide (20 mg/kg, Sigma-Aldrich), was administered intraperitoneally. Imaging was performed at least 10 min after administration.

**Figure S1**

**Figure S1. Cross-correlation analysis during the non-stimulation baseline and stimulation periods**

Cross-correlation analysis was performed on the time courses of GCaMP fluorescence intensity and arteriolar diameter changes over 3-s windows immediately before and after the onset of pinch stimulation (Non-stimulation baseline and During stimulation, respectively). Time lag indicates the time by which GCaMP fluorescence changes preceded arteriole diameter changes. Correlation coefficients are expressed as the median and interquartile range. The open triangle indicates a time lag of 2.37 s [r = 0.27 (IQR, 0.071–0.49); p = 0.015, tested by a one-sample t-test].

**Figure S2**

**Figure S2. Relationship between periarteriolar cholinergic varicosity Ca²⁺ signals and arteriolar diameter changes with and without autonomic ganglionic blockade**

Relationship between the peak amplitudes of GCaMP fluorescence intensity and arteriole diameter changes (ΔF/F(%) and Δ%, respectively) during pinch stimulation. Black circles indicate trials without hexamethonium, and light blue circles indicate trials after systemic administration of hexamethonium bromide (20 mg/kg, intraperitoneally). The solid line represents the linear regression for the combined data with and without hexamethonium (*R*² = 0.63, *p* = 0.0003).
